# Comparison between methylprednisolone infusion and dexamethasone in COVID-19 ARDS mechanically ventilated patients

**DOI:** 10.1186/s43162-022-00113-z

**Published:** 2022-02-15

**Authors:** Mohammed Abdel Monem Saeed, Alaa Hussein Mohamed, Ahmed Hassan Owaynat

**Affiliations:** 1 Critical Care Medicine Department, Faculty of Medicine, Helwan University, Cairo, Egypt; 2 Clinical Pharmacy Department, Helwan University Hospitals, Cairo, Egypt; 3 Nursing Department, Helwan University Hospitals, Cairo, Egypt

**Keywords:** COVID-19 pneumonia, ARDS, Mechanical ventilation, Steroids

## Abstract

**Background:**

Coronavirus disease 2019 (COVID-19) causing severe acute respiratory distress syndrome caused by coronavirus 2 (SARS-CoV-2) still has no solid effective therapy.

From previous studies, dexamethasone has led to a decrease in mortality in patients who required oxygen supplementation mainly invasive mechanical ventilation; at the same time, it is unknown if another corticosteroid can be effective when used and what is the optimal dose and its duration, to achieve improvement in clinical outcome.

The cornerstone of the study was to compare the differences in clinical outcome and laboratory results in intensive care patients with SARS-CoV-2 pneumonia treated with dexamethasone 6 mg/day: doses versus those treated with methylprednisolone 2 mg/kg/day infusion.

**Materials and methods:**

A prospective cohort study with a survival analysis of 414 patients diagnosed with severe COVID-19 pneumonia confirmed by polymerase chain reaction, for SARS-CoV-2 according to the Berlin definition of ARDS, who were admitted in the intensive care unit in the Helwan University Hospitals; the duration is from June 2020 till October 2021.

Patients included in the study were mechanically ventilated with radiological confirmation of pneumonia by chest tomography; patients were included in the study according to the Berlin definition of ARDS and met the inclusion criteria of the study; 222 patients were treated with methylprednisolone infusion with a dose of 2 mg/kg/day versus 192 patients treated with dexamethasone 6 mg/day; both groups were treated for 10 days and were mechanically ventilated; the clinical out come and differences in the laboratory results were evaluated during the 10-day course for each group.

**Results:**

Four hundred fourteen patients had COVID-19 pneumonia, diagnosed and confirmed by ground glass opacities in chest tomography and arterial partial pressure of oxygen/inspired oxygen and fraction of inspired oxygen (P/F ratio) less than 300.

Two hundred twenty-two patients received methylprednisolone infusion at a dose of 2 mg/kg/day, and 192 patients received dexamethasone 6 mg daily; both groups were treated for 10 days.

Inflammatory markers for cytokine storm were improved in the methylprednisolone group in comparison to the patients who were given dexamethasone when comparing the on-admission markers to the results of the inflammatory markers after 10 days, like ferritin after 10 days in methylprednisolone group 292.26 ± 330.10 versus the dexa group 648.10 ± 329.09 (*p* value < 0.001).

D-dimer in the methylprednisolone group was 1301.75 ± 1515.51 versus 2523.78 ± 843.18 in the dexa group (*p* value < 0.001); CRP was 49.65 ± 19.91 in the methylprednisolone group versus 100.54 ± 36.75 (*p* value < 0.001) in the dexa group; LDH after 10 days in methylprednisolone group was 345.09 ± 128.31, and in the dexa group, it was 731.87 ± 195.09 (*p* value < 0.001); neutrophil to lymphocyte ratio (N:L ratio) after 10 days of treatment in the methylprednisolone group was 17.27 ± 5.09 versus 26.68 ± 7.19 (*p* value < 0.001) in the dexa group; also, the length of stay was shorter in the methylprednisolone group (7.33 ± 1.71) versus in the dexa group (19.43 ± 5.42) (*p* value < 0.001), together with mechanical ventilation MV days which are 3.82 ± 1.14 in the methyl group versus 16.57 ± 4.71 in the dexa group (*p* value < 0.001).

Also, the radiological findings are improved in the methyl group (20.3%) versus the dexa group (73.4%) with *p* value < 0.001, and discharge from ICU in the methyl group was 79.7% versus 26.6% in the dexa group with *p* value < 0.001.

**Conclusions:**

Treatment of severe COVID-19 pneumonia, Patients who were mechanically ventilated with methylprednisolone infusion 2 mg/kg/day for 10 days versus dexamethasone 6 mg for 10 days showed a statistically significant improvement in the MV days and length of stay in the intensive care unit, together with the overall mortality and severity inflammatory markers of cytokine storm c-reactive protein (CRP), D-dimer, ferritin, LDH, and N:L ratio.

## Introduction

Severe acute respiratory syndrome caused by coronavrus-2 (SARS-CoV-2) was announced as a global pandemic by the World Health Organization (WHO) on March, 12, 2020; this was the third outbreak of beta coronaviruses in the twenty-first century, after the severe ARDS (SARS-COV) and Middle East respiratory syndrome (MERS-COV) causing a public health emergency worldwide [1–4]. This disaster was described first by the end of 2019 as a cluster of acute respiratory affections in Wuhan, Hubei province, China, which is, until the middle of January 2021, affecting over 93 million cases and leads to more than 2 million mortality cases in 218 countries worldwide [5].

Various aspects were impacted from the disease on the health care system and workers’ [6] diagnosis and dilemmas of overlapping with other diseases and management [7–9]. Also, it leads to mental and spiritual effect on the environment [10–12].

Lung affection is very fast, with high infectivity and no definitive effective medication, which contributed all to requirements to effective measures for COVID-19 management based on the progression of the disease and its pathogenesis [13, 14].

A lot of studies and researches have shared in the understanding of this disease and various empirical therapeutic managements, including the currently available and new antivirals and previous medication; an effective therapeutic management has not yet been received for severe COVID-19 patients [15, 16]. Cytokine imbalance was the main cause of organ dysfunction shown in the studies done on SARS [17].

In patients with COVID-19, a crucial period of chance for active management is considered when they start to deteriorate, where corticosteroids and other immunosuppressive agents are beneficial, as was previously in the experience of cases of SARS and MERS-COV [18–20].

**Fig. 1:**
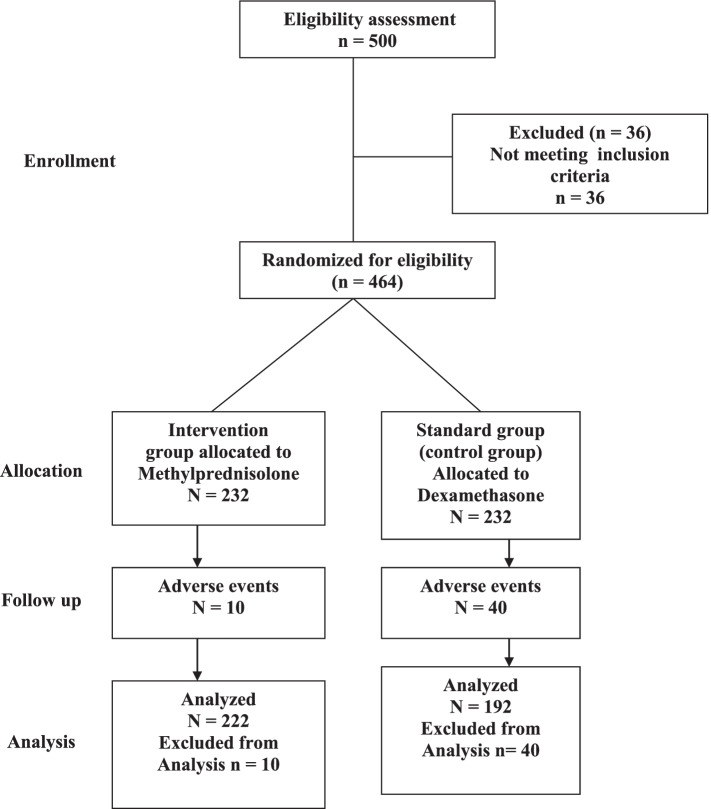
Flow diagram of a randomized clinical trial of methylprednisolone infusion vs dexamethasone in ARDS ventilated COVID-19 pneumonia patients

A major randomized clinical trial (RCT) was done in the UK, which reached to the conclusion that usage of low-dose dexamethasone in ventilated COVID-19 victims and to some extent in patients who require supplemental oxygen decreased the mortality [21] (Fig. 1). Meanwhile, the effect of the intermediate-acting corticosteroid, methylprednisolone, has been limited to recent date [22, 23].

Methylprednisolone is the main corticosteroid used in the intensive care unit (ICU) in most RCTS in the management of ARDS [24].

Methylprednisolone has high penetration to tissues in animal models than dexamethasone which is more effective for lung injury [24]. Previously methylprednisolone was effective on treating SARS disease [25, 26]. So, we proposed that methylprednisolone infusion may be more effective than other corticosteroids specifically dexamethasone. Depending on that information, we elicited a randomized control trial to detect the response to methylprednisolone infusion on the outcome of COVID 19 ARDS ventilated patients in comparison to the routinely used dexamethasone.

## Patients and methods

### Patients

Patients above 18 years old, who were admitted in the Helwan University Hospitals in the duration of June 2020 till October 2021 with COVID-19 pneumonia ARDS ventilated patients according to the Berlin definition protocol and confirmed by a real-time PCR, were included.

The excluded patients were those who are pregnant, who have uncontrolled diabetes mellitus (DM) and uncontrolled hypertension, who had previously been treated with steroids for any reason or any contraindications of steroid usage and immunodeficiency deficiency problems, and who are not fitting the definition of ARDS according to the Berlin protocol.

### Study design

Our research is a triple-blind RCT. Patients included in the Helwan University Hospitals in the duration of June 2020 till October 2021 were randomly allocated to receive a 10-day course of methylprednisolone at a dose of 2 mg/kg/day infusion or dexamethasone 6 mg with the standard care [20, 27]. Random assembly was done using the block randomization method.

During the study, the cases remained obscured. The patient, physician, and analyzer in the two groups did not have access to the randomization list and the type of administered drug (triple blind).

The first group received methylprednisolone infusion at a dose of 2 mg/kg/day and tapered to half a dosage every 5 days.

The methylprednisolone group was planned to be excluded from the start in any patient who faced severe elevation of systolic blood pressure > 180 mmHg and/or diastolic blood pressure > 120 mmHg or uncontrolled blood sugar which needs insulin infusion more than 5 IU/h to decrease blood glucose less than 180 mg/dL, for patients with type 2 diabetes mellitus.

The other group was randomized to receive 6 mg of dexamethasone intravenously for 10 days.

### Statistics

The collected data was revised, coded, tabulated, and introduced to a PC using the Statistical Package for Social Sciences (SPSS 20).

Data was presented, and suitable analysis was done according to the type of data obtained for each parameter.

i Descriptive statistics:
  1. Mean, standard deviation (± SD) and range for numerical data
  2. Frequency and percentage of non-numerical data
ii Analytical statistics:
  1. Student’s *T* test was used to assess the statistical significance of the difference between two study group means
  2. Chi-square test was used to examine the relationship between two qualitative variables

## Clinical and laboratory monitoring

All patients were evaluated for demographic features, underlying disease, P/F ratio, respiratory rate, and routine physical exams together with the laboratory and radiological investigation and outcome after 10 days from initiating the ventilatory parameters.

The treatment is by either methylprednisolone infusion or dexamethasone 6 mg.

## End points

The primary end points were the clinical, laboratory, and radiological status after 10 days of enrollment.

The secondary end points were for mortality, weaning from mechanical ventilation, and length of stay after 10 days of starting the active management.

## Ethical approval

The study was approved by the ethical committee of faculty of the Medicine Helwan University. It was done according to the local regulatory requirements, Good Clinical Practice (GCP), and the Declaration of Helsinki [28].

## Results

Four hundred fourteen patients were included in our clinical trial with 222 receiving methylprednisolone infusion at a dose of 2 mg/kg/day versus 192 receiving the standard treatment with dexamethasone 6 mg.

As mentioned in Table 1, there was no statistical significant variation between the two groups based on demographic features.

**Table 1:** Demographic data in the intervention group methylprednisolone infusion and dexamethasone group at baseline (*N* = 414)

		*N*	%	*p* value
Gender	Male	285	68.8%	0.55
	Female	129	31.2%	
Steroids	Methylprednisolone 2 mg/kg/day	222	53.6%	0.62
	Dexamethasone 6 mg/day	192	46.4%	

Table 2 shows the comparison of cytokine storm markers on admission and on the 10th day post initiating the definitive treatment methylprednisolone infusion versus the dexamethasone group (standard treatment dexamethasone 6 mg). In the table, on admission, the cytokine inflammatory markers, ferritin, D-dimer, CRP, LDH, and N:L ratio all were statistically insignificant when compared the definitive methylprednisolone versus the standard group with the exception of the D-dimer and LDH.

**Table 2:** Comparison of cytokine storm markers on admission and on the 10th day of treatment

	**Methylpred 2 mg/kg/day**		**Dexa. 6 mg**		***T*-test**	
	**Mean**	**SD**	**Mean**	**SD**	***t***	***p* value**
Ferritin admission	707.51	328.81	741.18	346.99	− 1.01	0.312
Ferritin + 10 days	292.96	330.10	648.10	329.09	− 10.93	< 0.001
D-Dimer admission	2657.05	1091.49	2997.62	982.75	− 3.34	0.001
D-Dimer + 10 days	1301.75	1515.51	2523.78	843.18	− 9.92	< 0.001
CRP admission	114.80	39.96	115.91	42.08	− 0.27	0.784
CRP + 10 days	49.65	19.91	100.54	36.75	− 17.14	< 0.001
LDH admission	782.55	282.57	857.69	230.84	− 2.98	0.003
LDH + 10 days	345.09	128.31	731.87	195.09	− 23.43	< 0.001
N:L ratio admission	30.36	8.20	30.85	8.28	− 0.60	0.551
N: L ratio + 10 days	17.27	5.09	26.88	7.19	− 15.47	< 0.001

Then, when comparing the ferritin level after 10 days in the methylprednisolone limb versus the dexamethasone limb, it shows a highly significant statistical difference *p* value < 0.001 (292.96 ± 330.10 vs 648.10 ± 329.09); also, D-dimer after 10 days shows a highly significant statistical difference *p* value < 0.001 when comparing both groups. CRP after 10 days was 49.65 ± 19.91 in the methylprednisolone group versus 100.54 + 3675 in the dexamethasone group (*p* value < 0.001). LDH after 10 days was 345.09 ± 128.31 in the methylprednisolone group versus 731.87 ± 195.09 in the dexamethasone group (*p* value < 0.001).

Neutrophil to lymphocyte ratio (N: L ratio) after 10 days was 17.27 ± 5.09 in the methylprednisolone group versus 26.88 ± 7.19 in the dexamethasone group (*p* value < 0.001).

Table 3 shows the days of MV in both groups together with the ICU length of stay, where there is dramatic clinical improvement in the methylprednisolone limb and weaning from mechanical ventilation with decreasing the MV days and ICU length of stay when compared to the dexamethasone limb. MV days was 3.82 + 1.14 in the methylprednisolone limb versus 16.57 + 4.71 in the dexamethasone limb (*p* value < 0.001).

**Table 3:** Mechanical ventilation days and length of stay in the two groups

	**Methylpred 2 mg/kg/day**		**Dexa. 6 mg**		***T*-test**	
	**Mean**	**SD**	**Mean**	**SD**	***t***	***p* value**
Los	7.33	1.71	19.43	5.42	− 29.68	< 0.001
MV days	3.82	1.14	16.57	4.71	− 36.57	< 0.001

Length of ICU stay was 7.33 + 1.71 in the methylprednisolone group versus 19.43 + 5.42 in the dexamethasone group (*p* value < 0.001).

Table 4 shows the radiological improvement by HRCT in the methylprednisolone group in comparison to the dexamethasone group.

**Table 4:** Radiological comparison between methylprednisolone group and dexamethasone group

		**Steroids**				**Chi-square test**	
		**Methylpred. 2 mg/kg/day**		**Dexa. 6 mg**			
		***N***	**%**	***N***	**%**	**Value**	***p*** ** value**
Radiology improvement HRCT	No	0	0.0%	192	100.0%	414	< 0.001
	Yes	222	100.0%	0	0.0%		

It was 222 in the methylprednisolone group versus 0 in the dexamethasone group, with *p* value < 0.001.

Table 5 shows the destination of the COVID-19 ARDS patients denoting the ICU discharge and patient death; 177 were discharged from the ICU in the methylprednisolone group (79.7%) versus 51 in the dexamethasone group (26.6%) with *p* value < 0.001.

**Table 5:** Destination of the studied groups

		**Steroids**				**Chi-square test**	
		**Methylpred. 2 mg/kg/day**		**Dexa. 6 mg**			
		***N***	**%**	***N***	**%**	**Value**	***p* value**
Destination	Dead	45	20.3%	141	73.4%	117.62	< 0.001
	Discharge	177	79.7%	51	26.6%		

As regards mortality, 45 patients out of 222 died in the methylprednisolone group (20.3%) versus 141 patients in the dexamethasone group (73.4%) with *p* value < 0.001.

## Discussion

The world population has faced a sudden stress by the emergence of COVID-19, and the virus continues to attack a lot of patients and destroy their lives with a high rate of death affecting those were infected; at the same time, physicians have to take treatment decisions without solid evidence during the management course of the COVID-19 patients. So, a lot of information was collected and started to explain the disease properties and definitive management; from these data, the researchers understood the role of the native immune reaction and the infectivity of the COVID-19 virus.

In our work, we aimed to evaluate the therapeutic effect of methylprednisolone as an additive management to the regular management protocol in the hospitalized ARDS COVID-19 ventilated victims in the ICU.

Our findings were correlated to the universal accepted corticosteroid treatment dexamethasone 6 mg based on the postulation that methylprednisolone has higher lung penetration [29, 30].

So, it can have a great immunosuppressive treatment in the management of COVID-19 ARDS ventilated patients and lead to better response of their respiratory hazards from the previous theory that methylprednisolone has higher lung penetration and can improve the respiratory complications; our data and results showed highly significant beneficial effects of the methylprednisolone infusion on the patients’ ICU stay and outcome as regards the clinical status, laboratory cytokine storm markers, weaning from mechanical ventilation, ICU length of stay, and also mortality rates in comparison to the patients who received dexamethasone 6 mg as we included a large sample size in our study.

The best response of corticosteroids in the treatment of COVID-19 was studied in many observational studies, as these agents are present worldwide, inexpensive, and not difficult to apply [31–33]. As there was no definitive response in other viral pneumonias regarding the safety and beneficial response of corticosteroids, the WHO with the start of the pandemic published recommendations against the routine use of steroids in managing patients with COVID-19 [26].

Meanwhile, it is well proven that glucocorticoid drugs are beneficial in decreasing the inflammatory storm by suppressing the pro-inflammatory gene expression and lessening the cytokine levels if used in the optimum time of disease progression [34]. Also, a considerable amount of studies reported an increase in mortality and prolongation of the time of viral eradication when using corticosteroids in MERS and influenza [20, 35]. At same time, regarding COVID-19 early researches, many variations according to the accepted and adequate dosage and way of giving steroids led to non-definitive results about the effectiveness of these drugs [19].

However, the coming studies have proven the great effect of methylprednisolone in patients a ttacked by COVID-19 in a randomized clinical trial done by Edalatifard et al.; the response of intravenous methylprednisolone pulse was evaluated [36], where patients who received methylprednisolone had a lower death rate and better survival time than the control group and, at the same time, little clinical findings like muscular pain, chest pain, cough, and gastrointestinal symptoms, in patients given methylprednisolone in comparison to the standard care group. Also, as regards the laboratory results, the methylprednisolone limb showed a decrease in the CRP level and thrombocytosis, although the dose and duration of methylprednisolone administration were different from our study, but their results are concordant with our results which revealed a dramatic beneficial effect in the methylprednisolone infusion at a dose of 2 mg/kg/day as regards the clinical status, radiologically by computed tomography HRCT, inflammatory cytokine storm markers, weaning from mechanical ventilation, and length of stay ICU as well as the ICU mortality.

Wang et al. had done a retrospective cohort study evaluating the victims affected by COVID-19 treated with low-dose methylprednisolone with short-term duration; they received 1–2 mg/kg/day methylprednisolone for 5–7 days; they experienced less hospital stay and decreased requirement for mechanical ventilation, but there was no statistical variation as regards death rate in comparison to the received dexamethasone 6 mg, the standard care, which are all in line with our results, but our results contains more sample size and also showed improvement in outcome as regards the immortality [37]. Many other studies showed a reduction in poor outcomes in patientstreated with methylprednisolone [38–40].

In our research, the two groups received corticosteroids; the control group received dexamethasone 6 mg, while the other limb received methylprednisolone 2 mg /kg/day for 10 days, halved after the 5th day; this leads to a better outcome, improving lung condition and decreasing inflammatory response and weaning from mechanical ventilation; this is mostly due to the better penetration of methylprednisolone in the lung tissue in comparison to dexamethasone; this leads to the obvious improvement in patients’ f ate; this as proposed by many studies shows better penetrance of methylprednisolone with lung tissue compared to other corticosteroids [41–43]. The only difference from the previous studies explained the relatively large doses of corticosteroid given that the estimated 6 mg of dexamethasone a day is equivalent to approximately 32 mg of methylprednisolone [44]; this affirms that the control group was taking an estimated dose of 0.5 mg/kg/day of methylprednisolone, based on 70 kg male, and we give in the methylprednisolone group a total dose of 2 mg/kg/day; this means four more times the equivalence of the dexamethasone 6 mg dose, and we are giving a more potent dose of methylprednisolone, but we have a large sample size of patients in comparison to the all previous studies, and we reached to the point that either due to the differences in dosage or medication, 2 mg/kg of methylprednisolone led to better net results in COVID-19 ARDS mechanically ventilated victims compared to 6 mg/day of dexamethasone.

Glucocorticoids can lead to some complications in patientswith COVID-19 like previous infection, immunosuppression, and hyperglycemia; latter researches revealed no prominent complications all through the study course, but in patients treated with methylprednisolone, hyperglycemia was more evident without any complications [36–38, 40].

Full dose of broad spectrum antibiotics and immune enhancers such as immunoglobulins should be used to improve the immune system in cases with complications [37].

One hundred seventy-five patients were studied by Yang et al. [45], with severe COVID-19, where he used methylprednisolone, which revealed to be as a protective factor against progression to critical disease done by the multivariate analysis (*p* value < 0.001; OR 0.054, 95% CI 0.017–0.173).

Also, a comparative observational was study done by Ruiz-Irastorza et al. [46], on patients with COVID-19 pneumonia, where he compared patients treated with methylprednisolone at a dose of 125–250 mg/day for 3 days with those who did not receive; the adjusted hazard ratios for death and or intubation for patients in the methylprednisolone group were 0.35 (95% CI 0.11 to 1.06, *p* value = 0.064) and 0.33 (95% CI 0.13 to 0.84, *p* value = 0.020), respectively.

Our study evaluated the improvement in clinical laboratory, radiologically by HRCT, mechanical ventilation weaning, ICU length of stay, and mortality in patients treated with high-dose methylprednisolone 2 mg//kg/day infusion versus the dexamethasone 6 mg/day.

All these positive findings were not reported previously in the vast of the studies done in the beginning of the pandemic; also, our study sample size had been done on a relatively large number of patients with ARDS COVID pneumonia; this improvement in the methylprednisolone limb corresponds to a dose-dependent effect of the corticosteroid which leads to a more significant reduction in the inflammatory response than patients who received dexamethasone 6 mg/day.

In the recovery trial, all markers of severity of COVID-19 cytokine storm were not available at baseline or follow-up.

Late studies also goes with our results and in its favor that severe ARDS ventilated patients when receiving methylprednisolone infusion at a dose of 2 mg/kg/day dramatically improved than those who received the standard dexamethasone 6 mg/day, which leads to faster weaning from mechanical ventilation and shorter duration of ICU stay [47, 48].

Our study had several positive findings as regards the improvement clinically, radiologically by HRCT, laboratory by cytokine storm markers, weaning from mechanical ventilations, ICU length of stay, and ICU mortality, and all goes in favor of the methylprednisolone infusion of a dose of 2 mg/kg/day over the dexamethasone 6 mg/day and applied on a relatively large sample size.

## Conclusion

Patient subjected to COVID-19 ARDS and mechanically ventilated and treated by intravenous infusion of methylprednisolone at a total dose of 2 mg/kg/day compared to dexamethasone 6 mg/day led to a dramatic improvement clinically, radiologically, laboratory, weaning from mechanical ventilation, ICU length of stay, and ICU mortality starting from the 5th to the 10th days.

## Data Availability

Available upon request.

## References

[1] Eurosurveillance Editorial Team (2020). Note from the editors: World Health Organization declares novel coronavirus (2019-nCoV) sixth public health emergency of international concern. Euro Surveill.

[2] World Health Organization (2020). WHO announces COVID-19 outbreak a pandemic. Available from: https://www.euro.who.int/en/health-topics/health-emergencies/coronavirus-covid-19/news/news/2020/3who-announces-covid-19-outbreak-a-pandemic. Accessed 7 Dec 2020

[3] World Health Organization (2020). Statement on the second meeting of the International Health Regulations (2005) Emergency Committee regarding the outbreak of novel coronavirus (2019-nCoV). Available from: https://who.int/news/item/30–01–2020statement-on-the-second-meeting-of-the-international-health-regulations-(2005)-emergency-committee-regarding-the-outbreak-of-novel–coronavirus-(2019-ncov). Accessed 11 Dec 2020

[4] World Health Organization (2019). Clinical management of severe acute respiratory infection when Middle East respiratory syndrome coronavirus (MERS-COV) infection is suspected; interim guidance. Available from: https://apps.who.int/iris/handle/10665/178529. Accessed 3 Jan 2021

[5] Worldmeter, COVID-19 Coronavirus Pandemic. 2020. J Availabe from: https://www.worldometers.Info/. Accessed 15 Jan 2021

[6] Sabetian G, Moghadami M, HashemizadehFardHaghighi L, Shahriarirad R, Fallahi MJ, Asmarian N (2021). COVID-19 infection among healthcare workers: a cross-sectional study in southwest Iran. J.

[7] Lotfi M, Sefidbakht S, Moghadami M, Iranpour P, Emami Y, Jafari SH, et al (2020) Introduction of a radiologic severity index for the 2019 novel coronavirus (COVID-19). 10.21203/rs.3.rs-47641/v1

[8] Shahriarirad R, Sarkari B (2020). COVID-19: clinical or laboratory diagnosis?. A matter of debate Trop Dr.

[9] Ashraf MA, Keshavarz P, Hosseinpour P, Erfani A, Roshanshad A, Pourdast A, et al (2020) Coronavirus disease 2019 (COVID-19): a systematic review of https://bmcifectdis.biomedcentral.com/articles/10.1186/s12879-021-06045-3. Prengnacy and the possibility of vertical transmission. J. Reprod Infertil 21(3):157–68 PMC7362089 32685412

[10] Mirahmadizadeh A, Ranjbar K, Shahriarirad R, Erfani A, Ghaem H, Jafari K (2020). Evaluation of students attitude and emotions towards the sudden closure of schools during the COVID-19 pandemic: a cross-sectional study. BMC Psychol.

[11] Shahriarirad R, Erfani A, Ranjbar K, Bazrafshan A, Mirahmadizadeh A (2021). The mental health impact of COVID-19 outbreak: a Nationwide survey in Iran. Int J Ment Health Syst.

[12] Erfani A, Shahriarirad R, Ranjbar K, Mirahmadizadeh A, Moghadami M. Knowledge, attitude and practice toward the novel coronavirus (COVID-19) outbreak: a population- based survey in Iran. Bull World Health Organ. 2020. 10.2471/BLT.20.256651

[13] Cheng ZJ, Shan J (2020). 2019 novel coronavirus: where we are and what we know published correction appears in infection. Infection.

[14] Young B, Tan TT, Leo YS (2021). The place for remdesivir in COVID-19 treatment. Lancet Infect Dis.

[15] Vetter P, Kaiser L, Calmy A, Agoritsas T, Huttner A (2020). Dexamethasone and remdesivir: finding method in the COVID-19 madness. Lancet Microbe.

[16] Shahriarirad R, Khodamoradi Z, Erfani A, Hosseinpour H, Ranjbar K, Emami Y (2020). Epidemiological and clinical features of 2019 novel coronavirus diseases (COVID-19) in the south of Iran. BMC Infect Dis.

[17] Huang C, Wang Y, Li X, Ren L, Zhao J, Hu Y (2020). Clinical features of patients infected with 2019 novel coronavirus in Wuhan. China Lancet.

[18] Mehta P, McAuley Dt, Brown M, Sanchez K, Tattersall RS, Manson JI (2020). COVlD-19: consider cytokine storm syndromes and immuno-suppression. Lancet.

[19] Stockman IJ, Bellamy R, Garner P (2006). SARS: systematic review of treatment effects. PLoS Med.

[20] Arabi YM, Mandourah Y, Al-Hameed F (2018). Corticosteroid therapy for critically ill patients with Middle East respiratory syndrome. Am J Respir Crit Care Med.

[21] Horby P, Lim WS, Emberson JR, Mafham M, Bell JL, Linsell L (2021). Dexamethasone in hospitalized patients with COVID-19. N Engl J Med.

[22] Williams DM (2018). Clinical pharmacology of corticosteroids. Respir Care.

[23] Sterne J, Murthy S, Diaz J, Slutsky A, Villar J, Angus D (2020). WHO rapid evidence appraisal for COVID-19 Therapies (REACT) working group. Association between administration of systemic corticosteroids and mortality among critically ill patients with COVID-19: a metaanalysis. JAMA.

[24] Annane D, Pastores SM, Arlt W, Balk RA, Beishuizen A, Briegel J (2017). Critical illness-related corticosteroid insufficiency (CIRCI): a narrative review from a Multispecialty Task Force of the Society of Crticial Care Medicine (SCCM) and the European Society of Intensive Care Medicine (ESICM). Intensive Care Med.

[25] Hui DS, Sung JJ (2003). Severe acute respiratory syndrome. Chest.

[26] Papamanoli A, Yoo J, Grewal P, Predun W, Hotelling J, Jacob R, et al (2020) High-dose methylprednisolone in nonintubated patients with severe COVID-19 pneumonia. Eur J Clin Investig:e13458. 10.1111/eci.13458 10.1111/eci.13458 PMC7744876 33219551

[27] COVID-19 Treatment Guidelines Panel. Coronavirus disease 2019 (COVID-19) treatment guidelines. National Institutes of Health. Available at https://www.covid19treatmentguidelines.nih.gov/. Accessed 1 Aug 2020 34003615

[28] Association WM (2013). World medical association declaration of Helsinki: ethical principles for medical research involving human subjects. JAMA.

[29] Meduri GU, Siemieniuk RAC, Ness RA, Seyler SJ (2018). Prolonged low-dose methylprednisolone treatment is highly effective in reducing duration of mechanical ventilation and mortality in patients with ARDS. J Intensive Care.

[30] Braude AC, Rebuck AS (1983). Prednisone and methylprednisolone disposition in the lung. Lancet.

[31] Fadel R, Morrison AR, Vahia A, Smith ZR, Chaudhry Z, Bhargava P (2020). COVID-19 management task force Early short-course corticosteroids in hospitalized patients with COVID-19. Clin Infect Dis.

[32] Alzghari SK, VSJJoCV A (2020). Supportive treatment with tocilizumab for COVID-19: a systematic review. J Clin Virol.

[33] Salvi R, Patankar P (2020). Emerging pharmacotherapies for COVID-19. Biomed Pharmacother.

[34] Darwish I, Mubareka S, Liles WC (2011). Immunomodulatory therapy for severe influenza. Expert Rev Anti-Infect Ther.

[35] Lansbury LE, Rodrigo C, Leonardi-Bee J, Nguyen-Van-Tam J, Shen LW (2020). Corticosteroids as adjunctive therapy in the treatment of influenza: an updated Cochrane systematic review and meta-analysis. Crit Care Med.

[36] Edalatifard M, Akhtari M, Salehi M, Naderi Z, Jamshidi A, Mostafaei S (2020). Intravenous methylprednisolone pulse as a treatment for hospitalized severe COVID-19 patients: results from a randomized controlled clinical trial. Eur Respir J.

[37] Wang Y, Jiang W, He Q, Wang C, Wang B, Zhou P (2020). A retrospective cohort study of methylprednisolone therapy in severe patients with COVID-19 pneumonia. Signal Transduct Target Ther.

[38] Corral L, Bahamonde A, de las Revillas FA, Gomez-Barquero J, Abadia-Otero J, Garcia- Ibarbia C, et al (2020) GLUCOCOV1D: a controlled trial of methylprednisolone in adults hospitalized with COVID-19 pneumonia. MedRxiv. 10.1101/2020.06.17.2013357-9

[39] Jeronimo CMP, Farias MEL Val FFA, Sampaio VS, Alexandre MAA, Melo GC et al (2020) Methylprednisolone as adjunctive therapy for patients hospitalized with COVID-19 (Metcovid): a randomized, double-blind, phase IIb, placebo-controlled trial. Clin Infect Dis:ciaa1177. 10.1093/cid/ciaa1177 10.1093/cid/ciaa1177 PMC7454320 32785710

[40] Nelson BC, Laracy J, Shoucri S, Dietz D, Zucker J, Patel N, et al (2020) Clinical outcomes associated with methylprednisolone in mechanically ventilated patients with COVID-19. Clin Infect Dis:ciaan63. 10.1093/cid/ciaa1163 10.1093/cid/ciaa1163 PMC7454332 32772069

[41] Braude A, Rebuck AJTL (1983). Prednisone and methylprednisolone disposition in the lung. Lancet.

[42] Hirano T, Homma M, Oka K, Tsushima H, Niitsuma T, Hayashi TJI (1998). Individual variations in lymphocyte-responses to glucocorticoids in patients with bronchial asthma: comparison of potencies for five glucocorticoids. Immunopharmacology.

[43] Vichyanond P, Irvin CG, Larsen GL, Szefler SJ, MRJJoa H (1989). Immunology C. Penetration of corticosteroids into the lung: evidence for a difference between methylprednisolone and prednisolone. J Allergy Clin Immunol.

[44] Mager DE, Lin SX, Blum RA, Lates CD, Jusko WJ (2003). Dose equivalency evaluation of major corticosteroids: pharmacokinetics and cell trafficking and cortisol dynamics. J Clin Pharmacol.

[45] Yang R, Xiong Y, Ke H, Chen T, Gao S (2020). The role of methylprednisolone on preventing disease progression for hospitalized patients with severe COVID-19. Eur J Clin Invest.

[46] Ruiz-Irastorza Guillermo, Pijoan Jose-lgnacio, Bereciartua Elena, Susanna Dunder, Jokin Dominguez (2020). Second week methyl-prednisolone pulses improve prognosis in patients with severe coronavirus disease 2019 pneumonia: an observational comparative study using routine care data. PLoS One.

[47] The Islamic Republic of Iran Medical Council (2020). Guide to diagnosis and treatment of COVID-19.

[48] World Health Organization (2020). WHO R&D Blueprint-Novel Coronavirus COVID-19 Therapeutic Trial Synopsis.

